# Level of Adoption of Hygiene Practices in Small-Scale Dairy Plants in Serbia

**DOI:** 10.3390/foods13152470

**Published:** 2024-08-05

**Authors:** Ilija Djekić, Nada Smigic, Zorana Miloradovic, Biljana Aleksic, Marijana Maslovarić, Rade Jovanović, Nataša Tolimir, Predrag Pudja, Jelena Miocinovic

**Affiliations:** 1 Faculty of Agriculture, University of Belgrade, Nemanjina 6, 11080 Zemun, Serbia; idjekic@agrif.bg.ac.rs (I.D.); nadasmigic@agrif.bg.ac.rs (N.S.); zorana@agrif.bg.ac.rs (Z.M.); pudja@agrif.bg.ac.rs (P.P.); 2 Dairy Institute, Smolucska 11, 11070 Belgrade, Serbia; biljana1972aleksic@gmail.com; 3 Institute for Science Application in Agriculture, Despota Stefana 68b, 11108 Belgrade, Serbia; mmaslovaric@ipn.co.rs (M.M.); rjovanovic@ipn.co.rs (R.J.); ntolimir@ipn.co.rs (N.T.)

**Keywords:** food safety, legislation, dairy products, short food supply chains

## Abstract

The main aim of this study was to analyze hygiene practices in small-scale dairy plants (SSDPs) in Serbia. A total of 60 plants were included in the research. A survey questionnaire used for SSDPs was designed to obtain the main information about hygiene practices they perform, as well as the data about the SSDPs, their production portfolio, and improvement plans. For the purpose of this study, a good hygiene practice score (GHPS) was calculated showing that the average score is 75%, spanning from 71.4% to 80.3% depending on the type of dairy plant. This study showed that the biggest challenges for small-scale dairy plants are associated with adequate labeling and external analysis of their dairy products, followed by record keeping and use of appropriate food contact materials. As expected, registered and approved SSDPs had higher GHPS scores and more information on their labels than those still in the approval or registration process. This study confirms the need for supporting this type of dairy producer to improve two main pillars of their business—the infrastructure for where they produce dairy products and awareness/knowledge of food safety legislative requirements. At the same time, approved processors are significantly underutilizing their processing capacity, which implies the need for both policy change consideration and educational initiatives. The policy changes should aim to align regulations with small-scale dairy processing realities.

## 1. Introduction

In the Republic of Serbia, milk production is estimated at around 1.5 billion L of raw milk per year [1]. Slightly below 50% of milk is processed by SSDPs (small-scale dairy plants) [2], producing different types of cheese and/or kajmak for home consumption [3,4]. SSDPs in Serbia are divided into two groups: “registered”, processing up to 200 L of milk per week, and “approved”, processing up to 5000 L of milk per week [5]. In the Republic of Serbia, about 13.5% of dairy products are sold through open markets, as most SSDPs prefer shorter distribution channels [6].

Short food supply chains typically involve a limited number of participants and often allow direct interaction between producers and consumers. Recognizing the importance of these short supply chains, the European Commission has integrated measures to support local food production and short supply chains into its rural development policy [7]. Specifically, for the production and sales of dairy products, this includes different stages, starting from the farm where the raw milk is produced, through to milk processing—especially cheese-making that often takes place in households—to transport, and finally, the direct sales to markets, which is usually carried out by the producers themselves. About 15% of all cheese production and sales take place within short supply chains, both in European Union (EU) member countries [8,9] and in the Republic of Serbia [4]. Short supply chains play an important role in the food–retail sector in the post-pandemic period [10], supported by their capacity to be pillars of sustainable supply chains [11]. The three-dimensional proximity advantages (physical, organizational, and social) enable selling at short distances with limited intermediaries at specific selling places such as at the farmer’s gate, roadside selling, or home deliveries [8]. The fact that about 50% of raw milk produced in Serbia is processed in households and SSDPs highlights the importance of this type of supply chain. In today’s competitive market, ensuring the safety and quality of products distributed in this way is an important prerequisite for maintaining and sustaining these existing short supply chains.

In both the European Union (EU) and the Republic of Serbia, the main responsibility for producing safe food lies with food business operators [12,13], regardless of their size. They are legally obliged to establish a food safety system based on the implementation of good hygiene practice (GHP), good manufacturing practice (GMP), and the application of the Hazard Analysis and Critical Control Points (HACCP) concept. While legislation mandates the application of HACCP for all food producers, except at the primary production level, small producers often face challenges in fully implementing these strict procedures. These challenges arise from limited human, financial, and technical resources [14] and can also result from a lack of clarity about the complex legal requirements [15,16,17]. To address these difficulties, the Republic of Serbia has adopted a set of regulations defining measures that allow flexibility, including certain exemptions, adaptations, and derogations from general and specific food hygiene requirements for dairy plants [5]. These derogations relate to infrastructure requirements and simplifications in the implementation of self-checking systems, provided they do not affect the safety of the final product [5,18].

Numerous scientific studies have addressed the concept of flexible hygiene measures, often examining the suitability and compatibility of national measures adopted at the EU level, as well as how individual EU member states have interpreted and implemented the flexible measures for small food producers [14,19,20]. It is of note that these tailored “flexibilities” are not exemptions that can compromise food safety in any way [21]. Several scientific studies have focused on the analysis of the microbiota of milk and dairy products produced by SSDPs, offering partial assessments of the hygiene conditions in production facilities [15,22,23,24,25,26].

Noor Hasnan et al. [27], in their review paper, reveal that inadequate design and facilities are the most prevailing discrepancies, highlighting rare on site supervision, a lack of resources, and poor knowledge as the main causes. A multiple-country survey of small food service establishments was analyzed from the perspective of food hygiene practices by Djekic et al. [28], highlighting hygiene and food preparation practices as the lowest ranked requirements, followed by the infrastructure of the premises. Food safety practices in SSDPs were the focus of research in Sri Lanka [29], comparing intensive and extensive farming systems. Their research emphasizes various malpractices and infrastructural issues, highlighting a need for additional training of farmers. Aleksic et al. [3] identified cold chain as an emerging topic associated with selling dairy products at open markets.

Besides the advantages of small businesses, small food producers have several challenges, food hygiene being the biggest challenge, especially when producing food of animal origin. There is currently a notable lack of information on the extent to which SSDPs in Serbia comply with hygiene requirements. This lack of data underlines the importance of conducting a comprehensive assessment in this regard. Such information is of utmost importance as it would enable the establishment of a set of measures and educational materials tailored to the specific needs and level of understanding of these producers. These tailor-made materials could play a crucial role in improving current practices and encouraging the introduction of higher hygiene standards in small-scale dairy production. The literature search revealed that the Serbian small-scale dairy industry has not been in research focus, and this was identified as a research gap by the authors. Therefore, the aim of this study was to assess the hygiene compliance of SSDPs in Serbia and pave the way for setting different improvement measures. From a scientific point of view, the working hypothesis was that hygiene compliance in SSDPs is not adequately addressed. In parallel, from a practical point of view, the working hypothesis is that SSDPs lack the knowledge to understand and implement hygiene requirements.

## 2. Materials and Methods

In total, 60 family farms engaged in dairy processing, distributed in 18 regions in the central part of Serbia, participated in this research. SSDPs were selected based on recommendations given by agriculture advisors from the Agriculture Advisory Services of Serbia operating in these regions. This ensured that the selected SSDPs would let us visit their premises and answer the questions.

The field survey was conducted from March to October 2022. The questionnaire used for SSDPs was designed to obtain the main information about hygiene practices they perform. In parallel, the questionnaire covered data about the SSDPs, their production portfolio, and improvement plans. Questions were selected and designed in accordance with the requirements of the Government Regulation (2017). The co-authors had three joint sessions to clarify the questions (from draft to final version). At the final session, the Delphi method was applied, stimulating and synthesizing the opinions among co-authors. Use of this iterative technique elicits experts’ knowledge and enables achieving consensus [30]. As a result, there were no holdouts for the final version of the questionnaire. Two types of questions were used, giving SSDPs the opportunity to answer yes/no or multiple-choice answers. Regarding the yes/no questions, the authors did not include an alternative to yes/no (such as “I don’t know” or “no opinion”) to avoid non-responses and to have the respondents clearly share their opinion. This is in line with the opinion of Krosnick et al. (2002), that the inclusion of “I don’t know” and/or “no opinion” does not improve the quality of data [31].

The research was performed as face-to-face interviews so that the farmers had the opportunity to clarify questions and add personal information and remarks. Four of the co-authors, with previous experience in similar studies, performed the field survey. Prior to commencing the interviews, one training session was organized to clarify how to perform the field survey.

The questionnaire was divided into several parts. The first part of the questionnaire referred to the general data on SSDPs, with questions regarding the type and quantity of processed milk, categories of products, products’ placement on the market, and the SSDP’s legal status. The following sections were related to production practices, facility/premises for milk processing and products’ storage, quality control, equipment for the preparation and processing of milk, waste disposal, packaging and labeling, awareness about legislation and food safety and quality standards, as well as family farm plans in terms of dairy production.

In total, all 60 representatives from the selected SSDPs fully answered all the questions, as displayed in Table 1.

### Data Processing

For each small-scale dairy plant that participated in this research, an overall good hygiene practice score (GHPS) was calculated by dividing the sum of positive answers (yes) with the total number of questions. The GHPS was analyzed using an analysis of variance (ANOVA) with Duncan’s post hoc test for comparing in-between scores of different characteristics of SSDPs.

Multiple-response questions and demographic characteristics (type of SSDP, type of milk processed) were subject to correspondence analysis. Statistical significance was set at *p* < 0.05. Statistical software used were Microsoft Excel 365, SPSS Statistics 23, and Minitab 17.

## 3. Results and Discussion

Table 1 shows the main characteristics of the sample. In accordance with the Serbian legislation, there are two categories of SSDPs—“registered” SSDPs, which encompass households processing operations capable of handling up to 200 L of milk per week from their own farm and marketing their products locally (at the farm, local green markets, and local retail outlets); and “approved” SSDPs, which pertains to small dairy facilities equipped to process up to 5000 L of milk per week, sourced from their own farms or neighboring ones, and authorized to sell their products throughout Serbia [5].

Out of 60 SSDPs that participated in this study, 24 (40.0%) are “approved” by the Veterinary Directorate of the Republic of Serbia, 7 (11.7%) are “registered” as SSDPs, and 29 (48.3%) are “in the process” of registration at the moment of interviewing. The majority of participants in this study process cow’s milk (85%), while the rest process goat and sheep’s milk (9%).

SSDPs mainly produce cheese and kajmak and sell it along with raw milk at open markets [3]. This type of short cheese supply chain is utilized in 15% of the total cheese production in Serbia [32]. The most popular type of cheese in Serbia is white brine cheese [33,34], which corresponds to cheese habits in this part of the world, i.e., south-east and eastern Europe and parts of the Middle East [32]. For producing this type of cheese, fresh cow’s milk—either without heating or heated up to 30 °C—is used, with the rennet then added to the (heated) milk to coagulate up to 6 h, depending on the region and tradition [35]. Kajmak is a crust layer of milk that is created as a result of cooling boiled milk [36]. The product is rich in aggregated milk fat and proteins [37]. Serbian consumers are very fond of kajmak, especially from SSDPs, as it is considered a traditional dairy product [38]; it is consumed fresh or ripened [36].

However, in consideration of the food safety risk, hygiene throughout this short supply chain is of utmost importance [39]. As for the whey produced by SSDPs, it is either used for feeding animals (on the farm), sold, or further processed.

While the Government Regulation of 2017 permits a substantial difference in the weekly milk processing limits for registered and approved small-scale dairy processors (200 L and 5000 L, respectively), the actual disparity in practice is comparatively modest, with quantities standing at 600 L and 1040 L, respectively. In the case of registered SSDPs, this indicates a non-compliance issue as they are operating beyond the specified limit. At the same time, approved processors are significantly underutilizing their processing capacity. These observations imply the need for both policy change consideration and educational initiatives. The policy changes should aim to align regulations with small-scale dairy processing realities, while education initiatives should empower processors with the knowledge and resources needed to operate efficiently and in compliance with the law.

Food of animal origin is sensitive to microbial (cross) contamination throughout the supply chain, from the animal to the consumer. These food-borne outbreaks may cause various types of diseases, from diarrhea to severe health problems [40]. However, as pointed out by Korale-Gedara et al. [29], most food safety issues associated with milk are preventable by targeting two GHP pillars—hygiene practices and infrastructure.

Table 2 displays the answers to 36 different good hygiene practices (GHP) requirements expected to be found in SSDPs. In general, the lowest scores (below 50%) were found regarding the labeling of the product (25.0%), use of stainless-steel workbenches (40.0%), external control of product (45.0%), and record-keeping (46.7%).

The widespread lack of food labels among small-scale dairy producers in Serbia, as also shown in our previous study [3], highlights a critical problem that requires immediate attention. As there is a mandatory requirement to label both packaged and unpackaged dairy products with essential information such as storage conditions and shelf life [5,41], the fact that a staggering 75% of producers surveyed do not include this information on their products is extremely worrying. These findings not only threaten consumer safety but also highlight the urgent need for regulators to enforce compliance and raise awareness of the pivotal role of food labeling in ensuring transparency and informed consumer choices. Even for small producers, there are legal requirements for having adequate food labels, especially when sold on open markets as both types of SSDPs can [3].

All equipment and utensils used in the facility must be designed and constructed to be clean, durable, and easily maintained. In addition, any surface that comes into direct contact with food must have properties such as corrosion resistance, non-toxicity, resistance to various environmental conditions during use, and the ability to protect food from potential sources of contamination. Although stainless steel is the most important and recommended material for food contact surfaces [42], it is worth noting that small-scale dairy producers may have some flexibility in using alternative materials for specific purposes, such as shelves, tools, presses, and molds, especially in the production of cheese with traditional characteristics [5]. Nevertheless, our research findings highlight that only 40% of participating small dairies use stainless-steel workbenches. Given the potential risks of cross-contamination and the operational challenges associated with materials other than stainless steel, it is essential to raise awareness and emphasize the importance of small dairies adhering to these guidelines.

Our results show that a total of 45% of the small-scale dairy producers (SSDPs) that participated in our study have results of external product control, while only 20% of SSDPs belong to the subgroup that is currently going through the registration process. The analysis of dairy products should primarily be used to verify strict compliance with hygiene standards, thus promoting a preventive approach to food safety, rather than to use the results of the analysis as the only proof of food safety performance, which is a corrective approach. In particular, those SSDPs that are in the middle of the registration process will be required to perform such analyses in order to obtain approval [5], and it is therefore reasonable to expect a higher compliance rate in this subgroup. It is important to emphasize that food safety regulations explicitly assign responsibility to food producers for ensuring the safety of their products [43]; however, flexible measures for SSDPs do not define the frequency of such controls. An interesting approach for street food, as another type of small-scale food producer, was proposed by [44], involving different stakeholders to formulate food safety strategies for small-scale producers [45] and allocating resources to control and improve the food safety of small premises and their products [46].

Medium scores (between 50% and 70%) were revealed for 13 requirements, associated with the use of cooling vats (51.7%), any means of milk control (55.0%), awareness of the HACCP concept (58.3%), use of production/technological procedures (60.0%), and awareness of food labeling legislation (63.3%). Although HACCP is not a mandatory requirement for SSDPs, scholars discuss the pros and cons of implementing HACCP in small-scale food businesses. However, most SSDPs confirm that the potential HACCP benefits of the near future are outweighed by the constraints of current business, as outlined by Taylor [47]. The main constraints in implementing food safety systems in Serbian small dairy companies are correlated with a lack of human and financial resources [48]. In parallel, several infrastructural issues scored medium scores, such as avoiding cross-contamination by separating raw materials and final products (63.3%); the condition of the walls (63.3%) and floors (66.7%) use of windows protective nets (66.7%); and types of food contact surfaces (70.0%).

Significant differences were found between small-scale dairy producers (SSDPs) in the process of registration and those already approved or registered (Table 2), particularly in the level of compliance with requirements relating to floor and wall materials, use of stainless-steel workbenches, external product control analysis, and knowledge of HACCP and labeling requirements (*p* < 0.05). These discrepancies are somewhat to be expected, considering that manufacturers in the initial stages of registration or approval may still be in the process of familiarizing themselves with the full range of legal hygiene requirements. Meeting these obligations often requires additional time and resources, including facility adjustments, structural improvements and investments, and infrastructural refurbishment, all of which contribute to this observed compliance discrepancy.

**Figure 1:**
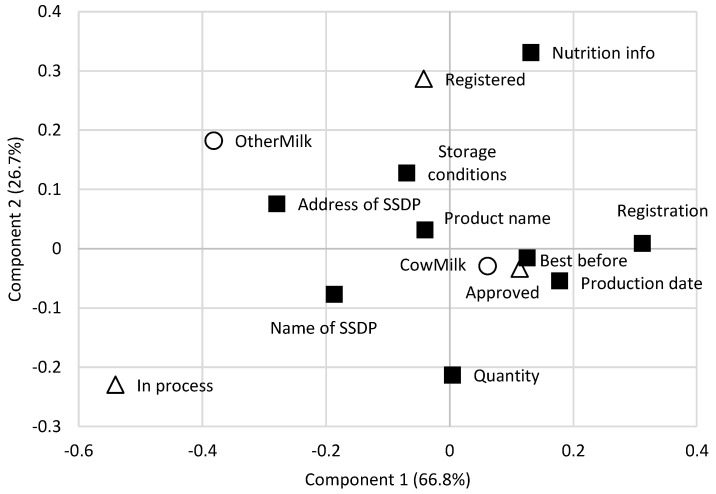
Multiple responses to “which data do you have on you labels?” The biplot displays the results of the correspondence analysis based on the data collected from 60 SSDPs. Legend of symbols: ■ label information; ◯ type of milk; △ type of SSDP. SSDP—small-scale dairy plant.

As below half of the SSDPs have any food labels, Figure 1 shows what type of information is displayed on their dairy products. The correspondence analysis shows that the first two dimensions explained 93.5% of the variance in the initial contingency data. It confirms that approved and registered processors of cow’s milk have more information on their labels, opposed to producers of dairy products with other types of milk (they only have the name and address of the producer). In parallel, as a rule of thumb, the results farther from the origin (intersection of the two components) are more discriminating. The “in process” SSDPs are highly discriminating SSDPs opposed to approved/registered SSDPs. Similarly, this applies to the “nutrition info.” as part of the label information. Opposed to this, results near the value “0” show that the product name, storage conditions, address/name of the SSDP, and date do not differentiate among the SSDPs.

The main role of food labels is to provide information to consumers and support their purchasing and/or consuming decisions [49]. The above-mentioned distinction of labeling information is in concurrence with two types of preferred information by consumers—“who” made the product and “what” is the basic nutritional information [50]. Serbian food labeling legislation [41] is fully aligned with EU legislation [51]. Consumer protection law pillars are founded on the rights of consumers in relation to the availability of products and truthful information, safety of the products, choice of conforming products, and legal protection [52].

Table 3 gives an overview of the good hygiene practice scores (GHPS) of all participants in our study and sheds light on a startling trend. It is noteworthy that the GHPS scores for the SSDPs were calculated separately, based on their registration or accreditation status and the locations where they sell their products, as well as their respective improvement plans. Not surprisingly, registered and approved SSDPs have higher GHPS scores than those still in the approval or registration process. This discrepancy is to be expected as registered and approved SSDPs have successfully gone through certain procedural steps, including veterinary inspections in the case of approvals. However, it is worth pointing out the unexpected observation that, in some cases, registered SSDPs outperform their licensed counterparts. This divergence can be attributed to historical nuances in the regulatory landscape. For decades prior to the adoption of the flexible measures [5], veterinary inspectorates had approved small operators and imposed less stringent requirements on them than on larger dairies. To give smaller producers access to the legal market, these inspections showed a degree of flexibility and considered the scale of production and distribution by small dairy producers. As a result, some SSDPs received their registration well before the new legislation came into force, without the need for subsequent renewal. At that time, the registration of milk producers with limited weekly milk production was not provided for in the legislation on veterinary requirements for dairies [53]. Therefore, all registered producers received their official registrations only recently, after 2017, while the approved SSDPs may have received their status some time ago. Consequently, the registered SSDPs are relatively new entrants to the dairy industry and may be better educated and aware of the current regulatory requirements. Consequently, it is advisable for the Ministry of Agriculture to re-evaluate and re-approve SSDPs that received their approval before 2017 to ensure alignment with evolving legal standards. In general, the majority of SSDPs sell their products on local markets, with only 26.7% selling throughout Serbia. Regarding improvement plans, the product portfolio is more in focus opposed to expanding the processing capacity of SSDPs, although 16.7% of SSDPs have no plans for any improvement.

**Figure 2:**
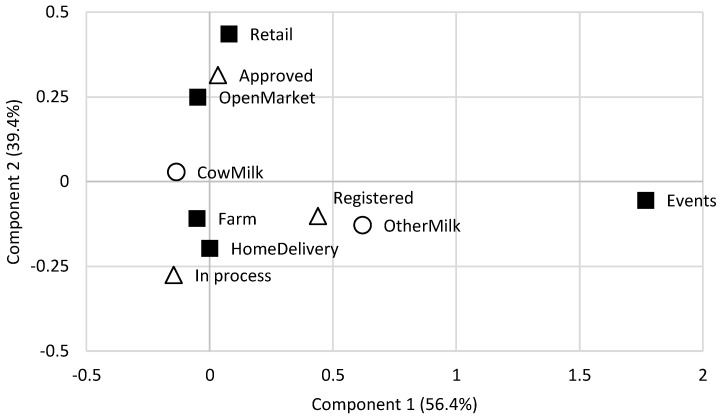
Multiple responses to “where do you sell your dairy products?” The biplot displays the results of the correspondence analysis based on the data collected from 60 SSDPs. Legend of symbols: ■ selling spot; ◯ type of milk; △ type of SSDP. SSDP—small-scale dairy plant.

Figure 2 depicts the trade channels of SSDPs. The correspondence analysis shows that the first two dimensions explained 95.8% of the variance in the initial contingency data and two patterns may be observed. Approved SSDPs are more focused on retail and open green markets opposed to registered SSDPs that mainly sell on their farms and provide home delivery, which is in line with the legislation in force [5]. Retail and events are the most discriminating selling spots. SSDPs using cow’s milk are not differentiated based on the data.

## 4. Conclusions

Considering that small-scale dairy producers (SSDP) predominantly operate within short supply chains, it is important that the challenges they encounter receive significant attention with proposed mitigative actions.

To improve the current understanding and compliance with regulations and standards, SSDPs should receive ongoing customized education on hygiene practices and food safety. They should be provided with adequate guidelines and manuals to help them transfer the generic legal requirements to their specific infrastructural capacities. For ongoing education, e-learning training could be an effective tool, along with online platforms where SSDPs can access information, guidelines, and updates at their convenience. Certification programs that recognize producers who have completed specific education and training requirements should be offered, to serve as a mark of quality and compliance.

Ongoing education for small-scale dairy producers is an investment in the long-term success and safety of this important dairy sector niche in Serbia. It would serve as a mechanism to uphold SSDPs’ knowledge, responsibility, and competence, thereby upgrading their capacity to consistently provide safe and high-quality products to consumers.

## Figures and Tables

**Table 1:** Main characteristics of the sample regarding milk and product type.

	In Process	Registered	Approved	Overall
Cow milk	25 (49.0%)	5 (9.8%)	21 (41.2%)	51 (85%)
Other type of milk	4 (44.4%)	2 (22.2%)	3 (33.3%)	9 (15%)
Raw milk processing	500 L/week	600 L/week	1040 L/week	725 L/week
Total	29 (48.3%)	7 (11.7%)	24 (40.0%)	60 (100%)
Type of Dairy Product Produced				
White brine cheese	26 (89.7%)	5 (71.4%)	22 (91.7%)	53 (88.3%)
Kajmak	12 (41.4%)	2 (28.6%)	9 (37.5%)	23 (38.3%)
Fresh cheese	8 (27.6%)	2 (28.6%)	8 (33.3%)	18 (30.0%)
Semi-hard cheese	2 (6.9%)	3 (42.9%)	7 (29.2%)	12 (20.0%)
Whey	3 (10.3%)	3 (42.9%)	4 (16.7%)	10 (16.7%)
Whey cheese	0 (0.0%)	1 (14.3%)	1 (4.2%)	2 (3.3%)

Legend: Data are presented as n (%); n represents the number of SSDPs; (%) represents their share in the sample. SSDP—small-scale dairy plant. Registered SSDPs—processing up to 200 L of milk per week; approved SSDPs—processing up to 5000 L of milk per week; in process—SSDPs in the process of registration by the Veterinary Directorate of the Republic of Serbia.

**Table 2:** Frequency of positive answers to different GHP requirements in three types of SSDPs.

	Fulfilled Requirement (%)			
GHP Requirement	In Process (*n* = 29)	Registered (*n* = 7)	Approved (*n* = 24)	Overall (N = 60)
Overall GHPS Score	71.4%	80.3%	77.7%	75.0%
Do you have a cooling vat?	44.8	71.4	54.2	51.7
Is the production area separated from the living area?	75.9	100	95.8	86.7
Is the processing area located separately from the animal housing facility?	96.6	100	91.7	95.0
Does the position, place and size of the facility/room allow conditional storage of food in terms of temperature?	79.3	100	91.7	86.7
Does the arrangement and equipment of the facility/premises allow appropriate maintenance, cleaning and disinfection?	93.1	100	87.5	91.7
Does the arrangement and equipment of the building/premises allow GHP in the handling of food, including protection against contamination, and especially pest control?	89.7	100	87.5	90.0
Are the floors made of impervious, non-absorbent, washable and resistant material that is easy to clean and, if necessary, disinfect?	65.5 ^a^	100 ^b^	87.5 ^a,b^	78.3 *
Do the floors of the rooms have an appropriate slope towards the sinks or drain openings that allows water not to be retained on the floor surfaces?	48.3 ^a^	85.7 ^b^	83.3 ^b^	66.7 *
Are the walls up to the ceiling lined with an impermeable, non-absorbent, washable and resistant material, with a smooth surface?	37.9 ^a^	85.7 ^b^	87.5 ^b^	63.3 *
Do the windows have protective nets that prevent the entry of insects?	55.2	57.1	83.3	66.7
Are all surfaces that come into contact with the food made of smooth, washable and non-toxic, corrosion-resistant material that is easy to clean and, if necessary, disinfect?	62.1	71.4	79.2	70.0
Are stainless steel workbenches used in the production of milk products?	24.1 ^a^	42.9 ^a,b^	58.3 ^b^	40.0 *
Are the floors, walls and surfaces in the processing room undamaged?	82.8	100	79.2	83.3
Is there a water supply and sewage outlet in the processing room?	86.2	85.7	100	91.7
Is there at least one hand washing facility in the processing room or in its immediate vicinity?	100	100	100	100
Is there a toilet nearby the production area?	93.1	85.7	83.3	88.3
Is there a changing area in the immediate vicinity?	96.6	85.7	83.3	90.0
Is the production of different products separated in time?	65.5	71.4	79.2	71.7
Is cleaning, washing and disinfection carried out between production stages?	79.3	85.7	79.2	80.0
Are raw materials and processed products in the same cooling area spatially separated and are all necessary measures taken to prevent possible contamination?	55.2	71.4	70.8	63.3
Does the water used in production have the quality of drinking water?	100	85.7	100	98.3
Is a clean scarf/cap worn during the stay in the milk processing area?	82.8	100	91.7	88.3
Do you wash your hands before entering the milk processing area?	96.6	100	87.5	93.3
Is a coat/apron used in the milk processing area, which is put away when leaving the processing area?	86.2	85.7	83.3	85.0
Are separate footwear used in the milk processing area?	75.9	85.7	79.2	79.3
Is there a standard production procedure, that is, a technological production procedure?	65.5	42.9	58.3	60.0
Is milk quality controlled?	48.3	85.7	54.2	55.0
Is product quality controlled (sent for analysis)?	20.7 ^a^	71.4 ^b^	66.7 ^b^	45.0 *
Are records kept on production conditions (raw material consumption and product quantity)?	37.9	57.1	54.2	46.7
Are tools, accessories and equipment made of smooth, easily washable and corrosion-resistant materials that are easy to clean and, if necessary, disinfect?	96.6	100	95.8	96.7
Is the waste material from the milk processing process regularly removed from the premises where it was created and temporarily placed in a dedicated container?	100	100	87.5	95.0
Does the manufacturer put a declaration (label) on the packaged product?	13.8	28.6	37.5	25.0
Is the producer familiar with the Regulation on small quantities of primary products that serve to supply consumers, the area for carrying out these activities, as well as deviations related to small entities in the business of animal products?	82.8	85.7	62.5	75.0
Is the producer familiar with the Regulation on the quality of raw milk (“Official Gazette of RS”, number 106/17)?	75.9	71.4	66.7	71.7
Are you aware of local regulations on labeling?	79.3	42.9	50.0	63.3 *
Have you heard about HACCP?	69.0 ^a^	28.6 ^b^	54.2 ^a^	58.3 *

* Statistical significance *p* < 0.05. Items denoted with different small letters are significantly different. SSDP—small-scale dairy plant. Registered SSDPs—processing up to 200 L of milk per week; approved SSDPs—processing up to 5000 L of milk per week; in process—SSDPs in the process of registration by the Veterinary Directorate of the Republic of Serbia.

**Table 3:** Characteristics of SSDPs and GHPS.

		In Progress (*n* = 29)		Registered (*n* = 7)		Approved (*n* = 24)		Overall (N = 60)	
		N (%)	GHPS	N (%)	GHPS	N (%)	GHPS	N (%)	GHPS
		29 (100.0%)	71.4%	7 (100.0%)	80.3%	24 (100.0%)	77.7%	60 (100.0%)	75.0%
Where are products sold?	Local market	26 (89.7%)	72.2%	4 (57.1%)	87.2%	14 (58.3%)	78.6%	44 (73.3%)	75.6%
	Entire Serbia	3 (10.3%)	64.9%	3 (42.9%)	71.2%	10 (41.7%)	76.5%	16 (26.7%)	73.3%
Improvement plans?	Improving dairy capacity	2 (6.9%)	60.8%	1 (14.3%)	67.6%	5 (20.8%)	88.1% ^a^	8 (13.3%)	78.7% ^a,b^
	Improving product portfolio	21 (72.4%)	73.2%	4 (57.1%)	79.0%	8 (33.3%)	76.3% ^a,b^	33 (55.0%)	74.7% ^a,b^
	Improving both	4 (13.8%)	74.3%	0 (0.0%)	-	5 (20.8%)	92.4% ^a^	9 (15.0%)	84.4% ^a^
	No plans	2 (6.9%)	58.1%	2 (28.6%)	89.2%	6 (25.0%)	58.6% ^b^	10 (16.7%)	64.6% ^b^

Legend: GHPS—good hygiene practice scores; SSDP—small-scale dairy plant; statistical significance *p* < 0.05. Items denoted with different small letters are significantly different within the group.

## Data Availability

The original contributions presented in this study are included in the article, further inquiries can be directed to the corresponding authors.

## References

[1] (2023). Statistical Yearbook of the Republic of Serbia for 2022.

[2] Veljković B., Koprivica R., Radivojević D., Mileusnić Z. (2018). Structure of exports and imports of milk and dairy products from Serbia. Acta Agric. Serbica.

[3] Aleksic B., Djekic I., Miocinovic J., Miloradovic Z., Savic-Radovanovic R., Zdravkovic N., Smigic N. (2023). The hygienic assessment of dairy products’ selling places at open markets. Food Control.

[4] Vlahovic B., Mugosa I., Puskaric A., Uzar D. (2018). Improving Cheese Production and Distribution.

[5] RS S.G. (2017). Regulation. Pravilnik o Malim Količinama Primarnih Proizvoda Koje Služe za Snabdevanje Potrošača, Području za Obavljanje tih Delatnosti Kao i Odstupanja Koja se Odnose na Male Subjekte u Poslovanju Hranom životinjskog Porekla.

[6] Petković G., Užar D. (2020). Marketing channels in value creation and delivery of cheese in the Republic of Serbia. Anal. Ekon. Fak. U Subotici.

[7] European Parliament, Council of the European Union (2013). Regulation (EU) No 1305/2013 of the European Parliament and of the Council of 17 december 2013 on support for rural development by the European Agricultural Fund for Rural Development (EAFRD) and repealing Council Regulation (EC) No 1698/2005. Off. J. Eur. Union.

[8] Malak-Rawlikowska A., Majewski E., Wąs A., Borgen S.O., Csillag P., Donati M., Freeman R., Hoàng V., Lecoeur J.-L., Mancini M.C. (2019). Measuring the Economic, Environmental, and Social Sustainability of Short Food Supply Chains. Sustainability.

[9] Worsfold D., Worsfold P.M., Griffith C.J. (2004). An assessment of food hygiene and safety at farmers’ markets. Int. J. Environ. Health Res..

[10] Ušča M., Tisenkopfs T. (2023). The resilience of short food supply chains during the COVID-19 pandemic: A case study of a direct purchasing network. Front. Sustain. Food Syst..

[11] Paciarotti C., Torregiani F. (2021). The logistics of the short food supply chain: A literature review. Sustain. Prod. Consum..

[12] (2004). Regulation (EC) No 854/2004 of the European Parliament and the Council of 29 April 2004 laying down specific rules for the organisation of official controls on products of animal origin intended for human consumption. Off. J. Eur. Union.

[13] (2011). Regulation on Vaterinary-Sanitary Conditions and General and Specific Hygiene Conditions on Food of Animal Origin.

[14] Ceballos L.A., Vercellino D., D’Errico V., Barzanti P., Decastelli L., Nicolandi L., Negro M., Ru G. (2020). Hazard perception and possibility of simplifying food safety management systems in small businesses in Piedmont region, Italy. Ital. J. Food Saf..

[15] Le S., Bazger W., Hill A.R., Wilcock A. (2014). Awareness and perceptions of food safety of artisan cheese makers in Southwestern Ontario: A qualitative study. Food Control.

[16] Buckley J.A. (2015). Food safety regulation and small processing: A case study of interactions between processors and inspectors. Food Policy.

[17] Walker E., Pritchard C., Forsythe S. (2003). Food handlers’ hygiene knowledge in small food businesses. Food Control.

[18] (2019). Guide for the Production and Processing of Milk in Objects of Small Capacity and for Producing Traditional Dairy Products.

[19] Mancuso A., Nicolandi L., Cabedo Botella L., Castoldi F., Jermini M., Wunsch A., Bompard C., Pisanello L. (2018). Analysis of Flexibility Principles in Food Safety in the EU and Consequent Assessment of the Notification Criteria (2004–2017). Eur. Food Feed Law Rev..

[20] De Boeck E., Jacxsens L., Kurban S., Wallace C.A. (2020). Evaluation of a simplified approach in food safety management systems in the retail sector: A case study of butcheries in Flanders, Belgium and Lancashire, UK. Food Control.

[21] Lawless J. (2012). The complexity of flexibility in EU Food Hygiene Regulation. Eur. Food Feed Law Rev..

[22] Carrascosa C., Millán R., Saavedra P., Jaber J.R., Raposo A., Sanjuán E. (2016). Identification of the risk factors associated with cheese production to implement the hazard analysis and critical control points (HACCP) system on cheese farms. J. Dairy Sci..

[23] Costa Dias M.A., Sant’Ana A.S., Cruz A.G., Faria J.d.A.F., Fernandes de Oliveira C.A., Bona E. (2012). On the implementation of good manufacturing practices in a small processing unity of mozzarella cheese in Brazil. Food Control.

[24] D’Amico D.J., Donnelly C.W. (2010). Microbiological quality of raw milk used for small-scale artisan cheese production in Vermont: Effect of farm characteristics and practices. J. Dairy Sci..

[25] Machado R.A.M., Cutter C.N. (2017). Sanitation indicators as a tool to evaluate a food safety and sanitation training program for farmstead cheese processors. Food Control.

[26] Rosengren Å., Fabricius A., Guss B., Sylvén S., Lindqvist R. (2010). Occurrence of foodborne pathogens and characterization of Staphylococcus aureus in cheese produced on farm-dairies. Int. J. Food Microbiol..

[27] Noor Hasnan N.Z., Basha R.K., Amin N.A.M., Ramli S.H.M., Tang J.Y.H., Aziz N.A. (2022). Analysis of the most frequent nonconformance aspects related to Good Manufacturing Practices (GMP) among small and medium enterprises (SMEs) in the food industry and their main factors. Food Control.

[28] Djekic I., Smigic N., Kalogianni E.P., Rocha A., Zamioudi L., Pacheco R. (2014). Food hygiene practices in different food establishments. Food Control.

[29] Korale-Gedara P., Weerahewa J., Roy D. (2023). Food safety in milk: Adoption of food safety practices by small-scale dairy farmers in Sri Lanka and their determinants. Food Control.

[30] Heiko A. (2012). Consensus measurement in Delphi studies: Review and implications for future quality assurance. Technol. Forecast. Soc. Chang..

[31] Krosnick J.A., Holbrook A.L., Berent M.K., Carson R.T., Michael Hanemann W., Kopp R.J., Cameron Mitchell R., Presser S., Ruud P.A., Kerry Smith V. (2002). The impact of “no opinion” response options on data quality: Non-attitude reduction or an invitation to satisfice?. Public Opin. Q..

[32] Aleksic B., Djekic I., Miocinovic J., Miloradovic Z., Memisi N., Smigic N. (2022). The application of Failure Mode Effects Analysis in the long supply chain—A case study of ultra filtrated milk cheese. Food Control.

[33] Miloradovic Z., Blazic M., Barukcic I., Font i Furnols M., Smigic N., Tomasevic I., Miocinovic J. (2022). Serbian, Croatian and Spanish consumers’ beliefs towards artisan cheese. Br. Food J..

[34] Bulajic S., Colovic S., Misic D., Djordjevic J., Savic-Radovanovic R.J., Ledina T. (2017). Enterotoxin production and antimicrobial susceptibility in Staphylococci isolated from traditional raw milk cheeses in Serbia. J. Environ. Sci. Health Part B.

[35] Vesković-Moračanin S.M., Mirecki S., Trbović D.K., Turubatović L.R., Kurćubić V.S., Mašković P.Z. (2012). Traditional manufacturing of white cheeses in brine in Serbia and Montenegro: Similarities and differences. Acta Period. Technol..

[36] Aleksic B., Djekic I., Smigic N., Miloradovic Z., Tomic N., Miocinovic J. (2023). Challenges in Evaluating Quality of the Serbian Traditional Dairy Product Kajmak. J. Food Qual..

[37] Miocinovic J., Miloradovic Z., Radulovic Z., Paunovic D., Trpkovic G., Radovanovic M., Pudja P. The composition and properties of Kajmak from different producers. Proceedings of the 22nd International Scientific-Expert Conference of Agriculture and Food Industry.

[38] Pudja P., Djerovski J., Radovanović M. (2008). An autochthonous Serbian product–Kajmak Characteristics and production procedures. Dairy Sci. Technol..

[39] Lahou E., Uyttendaele M. (2017). Growth potential of Listeria monocytogenes in soft, semi-soft and semi-hard artisanal cheeses after post-processing contamination in deli retail establishments. Food Control.

[40] Singhal P., Kaushik G., Hussain C.M., Chel A. (2020). Food safety issues associated with Milk: A review. Saf. Issues Beverage Prod..

[41] (2022). Ordinance on Labelling, Marking and Advertising of Food Products.

[42] EHEDG (2018). Hygienic design principles. EHEDG Guidelines.

[43] (2019). Food Safety Law.

[44] Contreras C.P.A., Cardoso R.d.C.V., da Silva L.N.N., Cuello R.E.G. (2020). Street Food, Food Safety, and Regulation: What is the Panorama in Colombia?: A Review. J. Food Prot..

[45] Haque I.T., Kohda Y. (2020). Understanding the impact of social determinants of health in street food safety: A qualitative study in Bangladesh. Int. J. Health Promot. Educ..

[46] Pilamala Rosales A., Linnemann A.R., Luning P.A. (2023). A Net-Map analysis to understand the roles and influence of stakeholders in street food safety—A study in Ecuador. Food Control.

[47] Taylor E. (2001). HACCP in small companies: Benefit or burden?. Food Control.

[48] Tomasevic I., Smigic N., Djekic I., Zaric V., Tomic N., Miocinovic J., Rajkovic A. (2016). Evaluation of food safety management systems in Serbian dairy industry. Mljekarstvo.

[49] Madilo F.K., Owusu-Kwarteng J., Parry-Hanson Kunadu A., Tano-Debrah K. (2020). Self-reported use and understanding of food label information among tertiary education students in Ghana. Food Control.

[50] Gracia A., de-Magistris T. (2016). Consumer preferences for food labeling: What ranks first?. Food Control.

[51] European Parliament, Council of the European Union (2011). Regulation (EU) No 1169/2011 of the European parliament and of the council of 25 October 2011 on the provision of food information to consumers, amending Regulations (EC) No 1924/2006 and (EC) No 1925/2006 of the European Parliament and of the Council, and repealing Commission Directive 87/250/EEC, Council Directive 90/496/EEC, Commission Directive 1999/10/EC, Directive 2000/13/EC of the European Parliament and of the Council, Commission Directives 2002/67/EC and 2008/5/EC and Commission Regulation (EC) No 608/2004. Off. J. Eur. Union.

[52] (2021). Consumers Protection Law.

[53] (2014). Regulation on Veterinary and Sanitary Conditions Regarding Hygiene of Animal Origin Food and Hygiene Conditions If Animal Origin Food RS.

